# Exposure to an aversive odor alters *Caenorhabditis elegans* physiology

**DOI:** 10.17912/micropub.biology.001198

**Published:** 2024-05-03

**Authors:** Joyobrata Sarkar, Kshitij Vashisth, Anubhuti Dixit

**Affiliations:** 1 Amity Institute of Neuropsychology and Neurosciences, Amity University, Noida, Uttar Pradesh, India

## Abstract

Perception of external cues is important for enhancing the fitness and survival of animals. However, the role of odor perception in regulation of longevity and health is incompletely defined. Here, we show that the exposure to an aversive odor 2-nonanone reduces life span, brood size, feeding rate, and increases lipid storage in worms. These effects are restored to normal levels in mutant worms lacking functional olfactory AWB neurons, suggesting a potential role of odor perception in the regulation of animal physiology and longevity.

**Figure 1. Effect of 2-nonanone odor exposure on C. elegans physiology:**
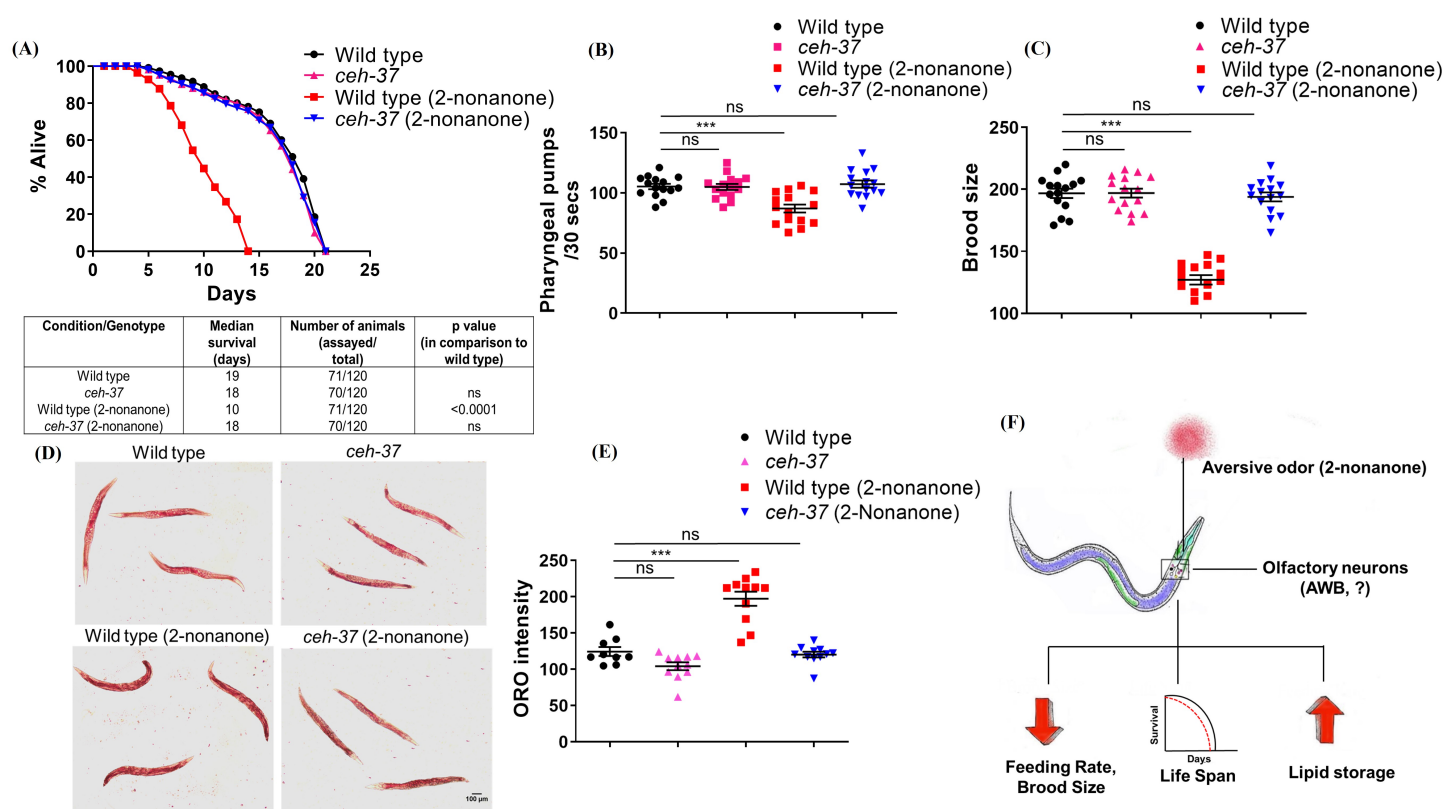
Kaplan-Meier survival curves and life span assay statistics of wild type and *ceh-37* animals exposed to 2-nonanone (dilution 1:50), (B) pharyngeal pumping rates of wild type and *ceh-37* animals in the presence and absence of 2-nonanone odor, (C) brood size of wild type and *ceh-37* animals in the presence and absence of 2-nonanone odor, (D) Oil-Red-O staining of lipid droplets (scale bar 100 µM), and (E) Quantification of lipid droplet stores in wild type and *ceh-37* animals in the presence and absence of 2-nonanone odor, (F) The perception of aversive odor 2-nonanone by sensory neurons (possibly AWB) causes decrease in feeding rate, life span, brood size and increase in lipid stores in *C. elegans* . *** = p < 0.001; ns= non-significant.

## Description

The ability to sense environmental chemicals exists in nearly all life forms, ranging from single-celled organisms to humans. Animals show distinct behaviors in response to different olfactory cues. They may show olfactory preference towards attractive odors such as food or aversion to harmful odors such as pungent chemicals. Thus, the olfactory function of an organism can act as a decisive factor for its survival under varied environmental conditions. Olfactory dysfunction is common in the aged population and is also the primary contributor to taste loss in the elderly. Studies based on animal models have shown that loss of olfactory receptors or neurons causes animals to live longer or shorter (Apfeld and Kenyon 1999; Alcedo and Kenyon 2004; Dixit and Bhattacharya 2021) . The nematode *Caenorhabditis elegans* has been considered one of the best models to study aging due to its short life span, known connectome, and conserved signaling pathways. *C. elegans* depends primarily on its chemosensory system to acquire information about its environment and can sense dozens of odors through several olfactory sensory neurons such as AWA, AWB, and AWC neurons (Troemel et al., 1997) . Previously it has been found that exposure to an attractive odor isoamyl alcohol increases the life span of worms (Kurino et al., 2017) while exposure to another attractive odor diacetyl shortens the food deprivation-induced longevity in *C. elegans* (Park et al., 2021) . Olfactory perception of food odor has been shown to regulate longevity through brain to gut signalling. Finger and colleagues have found that perception of food odor by AWC neuron promotes longevity through microRNA *mir-71* mediated regulation of protein turnover in the intestine (Finger et al., 2019) . Similarly, Zhang and coworkers have shown that recognition of food-associated odors by sensory neurons ADF and CEP shortens the dietary restriction induced life span by signalling to gut via octopamine, the mammalian homolog of norepinephrine (Zhang et al., 2021) . Another study by Miller and colleagues has shown that perception of attractive food odor by AWC neuron inhibits dietary restriction-mediated *fmo-2* induction in intestine and longevity (Miller et al., 2022) . However, effect of aversive odor on animals' health and life span is still unknown. In *C. elegans,* odorant 2-nonanone is known to elicit repulsive responses (Troemel et al., 1997) . In the current study, we tried to understand the effect of 2-nonanone odor on the life span and health span of *C. elegans* . We found that the wild type worms exposed to 2-nonanone odor live shorter as compared to unexposed worms ( Fig. 1A ). Reproductive decline and reduced pharyngeal pumping because of muscle weakening are hallmarks of aging in *C. elegans* . We assessed the effect of 2-nonanone odor on the feeding rate and brood size of worms. Results showed that exposure to 2-nonanone odor decreases feeding rate and brood size of wild type worms in comparison to unexposed worms ( Fig. 1B and C ). This suggests that the presence of aversive odor causes the early appearance of age-related phenotypes in worms. The olfactory sensory neurons AWB detect and mediate avoidance from 2-nonanone in *C. elegans* . *ceh-37* is an OTD/OTX family of homeodomain proteins required for the identity and olfactory function of AWB neurons (Lanjuin et al., 2003) . We investigated the effect of 2-nonanone odor on life span, feeding rate, and brood size of *ceh-37* mutant worms. We found that the absence of functional AWB neurons in *ceh-37* mutant reverses the 2-nonanone-induced decrease in life span, pharyngeal pumping, and brood size of worms up to wild type levels ( Fig. 1 A, B, and C ). The results suggest that perception of 2-nonanone odor by AWB neurons could be necessary for producing the negative influence of aversive odor on worm's physiology.

Next, we thought that the effect of 2-nonanone odor on feeding rate could be reflected on lipid stores levels as well. Therefore, we quantified the level of lipid stores in wild type worms exposed to 2-nonanone odor and found that 2-nonanone-exposed worms display around a 1.6-fold increase in lipid storage as compared to unexposed worms. In addition to this, the increased lipid stores have been restored to normal levels in the *ceh-37* mutant ( Fig. 1D and E ) indicating the possible role of olfactory neurons AWB in 2-nonanone odor-induced increase in lipid storage. Increased level of lipid stores in worms exposed to 2-nonanone odor reflects less utilization of stored energy. It suggests that the olfactory system modulates feeding behavior which in turn could affect fat deposits and obesity. However, decreased feeding rate and increased lipid stores in 2-nonanone exposed worms may not be causally related and could be two independent outcomes of 2-nonanone exposure. The observation of a recent study that recognition of butanone odor by AWC neurons alters lipid storage in worms without affecting eating behavior (Mutlu et al., 2020) indicates the same. Moreover, Mutlu et al. did not find any change in lipid storage when worms were exposed to 2-nonanone odor which is in contrast with our data. There could be three reasons for this discrepancy. First, they have exposed worms to 2-nonanone odor for short duration, i.e. 4 hours while we have given a continuous exposure of odor from L1 stage till day 2 when lipid stores assessment was done. Second, the concentration of 2-nonanone they have used is 2ul undiluted while we have used 10ul of 1:50 diluted odorant. Third, day 1 worms were used for lipid storage analysis by Mutlu et al. while we have used day 2 worms. Additionally, one of the reasons for increased lipid levels in 2-nonanone exposed worms could be reduced reproductive ability. A decrease in brood size of 2-nonanone-exposed worms may result in restricted fat distribution and a further increase in the level of lipid stores. The similar finding has shown that *rict-1* mutants are shorter-lived and have elevated fat content possibly due to shunting of calories away from reproduction or inadequate utilization of stored fat (Soukas et al., 2009) . Moreover, it has been shown that in stressed cells, lipid droplets maintain energy and redox homeostasis and protect against lipotoxicity by sequestering toxic lipids into their neutral lipid core (Jarc and Petan 2019) which further explains the elevated lipid levels in worms exposed to aversive odor 2-nonanone. Results of current work suggest that presence of functional AWB neurons might be mediating the harmful effects of 2-nonanone odor in *C. elegans* . The possible reason for this could be the overstimulation of AWB olfactory neurons in the presence of 2-nonanone odor which is leading to dysregulation of other physiological functions and shifting the equilibrium towards the detrimental side. Similar examples exist in literature where exaggerated olfactory sensitivity to threat in anxiety could be the cause of deleterious symptoms of anxiety (Krusemark and Li 2012) . We can speculate here that the neuronal response to aversive smell is creating an anxiety-like condition and associated symptoms such as loss of appetite and obesity in worms. However, further research is needed to validate this hypothesis. Moreover, conclusions of study are preliminary as we have not assessed the involvement of neurons other than AWB, in mediating the effect of 2-nonanone odor on *C. elegans* physiology. Since the *ceh-37* gene is expressed in other sensory neurons as well, fate of these neurons may be altered in *ceh-37* mutant (Lanjuin et al. 2003) . To the best of our knowledge, the current study shows for the first time that exposure to aversive odor 2-nonanone reduces the health and life span of animals, and this effect may be regulated by a pair of olfactory neurons ( Fig. 1F ).

## Methods

***C. elegans* strains and culture conditions**

*C. elegans* were cultured on nematode growth media (NGM) plates at 20°C on *E. coli* OP50 bacteria as a food source as per standard protocol (Brenner 1974) . *C. elegans* strains used are listed in the reagent table. *E. coli* was grown overnight in Luria Broth at 37°C. NGM agar plates containing 100 µg/ml streptomycin were seeded with 0.2 ml of overnight-grown bacterial culture. Plates were incubated at room temperature for 2-3 days before being used for experiments.

**Odor exposure**

All the experiments were performed at 20°C and in the presence of odorant 2-nonanone. A thin layer of NGM agar was added on the lids of NGM plates used for assays. During the preparation of NGM plates, media was poured in lids as well as plates. However, in the lid we poured less quantity of media (around 5-6 ml) which resulted in formation of thin layer. 10 ul of 2-nonanone, diluted (1:50) with ethanol, was added at two points inside the lid of NGM plate and plates were sealed with parafilm. Similarly, for controls, wild type worms were exposed to solvent ethanol. Worms were exposed to odorant or solvent starting from L1 stage and odor was replenished each day till the duration of experiment.

**Life span assay**

The life span of worms was assayed on 60 mm NGM plates seeded with *E. coli* culture. Wild type and mutant strains were exposed to odorant or solvent from L1 stage till the duration of experiment. Worms were synchronized to the young adult stage and transferred to fresh plates for life span assessment. The number of dead worms were counted each day and live worms were transferred to fresh NGM plates every day. Animals that crawled off the plate or exploded were included under the column ‘gone' and included in the data analysis. Graph was plotted in Graph Pad Prism software with its corresponding wild type and TD50 values (time when half of the population dies) were obtained for each strain. p values were calculated using the Log-rank (Mantel-Cox) method. A total of two replicates were performed for life span assay and more than 100 animals were used for each group in each replicate.

**Feeding rate**

The feeding rate was quantified by counting the pharyngeal pumping of synchronized well-fed day 2 adult worms. Wild type and mutant strains were exposed to odorant or solvent from L1 stage till day 2. Pharyngeal pumping was assessed by manually counting the number of pharyngeal contractions of worms in 30-seconds under a stereomicroscope. A total of three replicates were performed and 5-6 animals were assessed for each group in each replicate.

**Brood size**

Brood size was measured as reported elsewhere (Dixit et al., 2020) . Single late L4 stage worms of each wild type or mutant strain were kept on individual OP50-seeded plates and exposed to odorant or solvent till the duration of experiment. The animals were transferred to a new plate every 24 hours until they stopped producing progeny. All the plates containing progeny were incubated for 2 days and the number of worms were counted. Total brood size for each worm was determined by adding together the numbers of progeny produced by a single worm each day. A total number of 15 animals were assessed for each group. A total of three replicates were performed and 5-6 animals were assessed for each group in each replicate.

**Oil-Red-O staining and its quantification**

Lipid staining was performed by the Oil-Red-O staining method as described earlier (Soukas et al., 2009) . Wild type and mutant strains were exposed to odorant or solvent from L1 stage till day 2. Briefly, two days old, synchronized worms were washed twice with Phosphate Buffered Saline (PBS). The worms were then re-suspended in buffer containing an equal volume of PBS and 2XMRWB buffer with 2% paraformaldehyde (composition: 160 mM KCl, 40 mM NaCl, 14 mM Na _2_ EGTA, 1 mM Spermidine HCl, 0.4 mM Spermine, 30 mM Na PIPES at pH 7.4, 0.2% β-mercaptoethanol). The worms were then washed once in PBS, re-suspended in 60% isopropanol to dehydrate and 60% Oil-Red-O stain was added overnight. The next day worms were washed twice with PBST (0.1% Triton-X is added in PBS) and were mounted onto 2% agar pads and imaged at 10X magnification using a Magnus upright compound microscope. Images were quantified with Image J software (NIH, USA). A total of two replicates were performed and ORO intensity 5-6 animals was quantified for each group in each replicate.

**Statistical analysis**

Data are presented as mean ± SEM. Lifespan data were analyzed by using the log-rank (Mantel-Cox) test. p < 0.05 was considered significant. Other data were analyzed by using one-way ANOVA. The statistical software used for data analyses is Graph Pad Prism 9.0.

## Reagents

**Table:** 

**Strain**	**Genotype**	**Available from**
N2	Wild type	CGC
LJ1	*ceh-37 ( ok272 ) *	CGC
